# Can I Get a Witness? The Ethical Dimensions of Family Presence in Patient Suffering

**DOI:** 10.1002/hast.70037

**Published:** 2026-07-20

**Authors:** Jennifer Blumenthal‐Barby, Trevor M. Bibler, Holland Kaplan, Adam Omelianchuk, Joanna Smolenski

**Keywords:** doctor‐family relationship, surrogate decision‐making, clinical ethics, suffering, end‐of‐life decision‐making, family duty

## Abstract

For patients who are suffering, the bedside presence of a family member can provide comfort, and many people hold that there is moral value in being present with a conscious, suffering patient. Yet what is the moral significance of the absence of family members when a patient is minimally conscious or unconscious and not aware of their absence? Clinicians are often troubled when family members and surrogate decision‐makers who are able to spend a significant amount of time at an unconscious, seriously ill patient's bedside do not do so. Clinicians feel frustrated that they must bear the burden of witnessing the patient's actual or perceived suffering while the family escapes this burden and therefore appears to fail to uphold a duty to the patient. What are we to make of this point of view? What is the source of this frustration? What is the nature of the perceived duty? Is it morally defensible to request or require family members to be present with an unresponsive patient to bear witness to their actual or perceived suffering? If so, on what grounds?

## Article

It is generally recognized that being present with a conscious, suffering patient is morally valuable. The touch, words, or mere presence of a familiar friend or family member can provide great comfort to patients in the face of physical, emotional, or existential suffering. What, however, is the moral significance of the absence of family members^1^ when a patient is minimally conscious or unconscious and not aware of their absence?

This important question arises frequently in clinical practice. Several of us have received requests for ethics consultation or personally experienced distress surrounding an absent surrogate or other supportive party. Clinicians are often bothered when family members and surrogate decision‐makers can but infrequently do spend a significant amount of time at the bedside of an unresponsive^2^ and seriously ill patient. Part of what we hear in these cases is frustration that the clinical team must bear the burden of witnessing the patient's actual or perceived suffering continuously during the provision of care, while the family members or surrogates escape this burden and therefore appear to fail to uphold a perceived duty to the patient. What is one to make of this claim? What is the source of this frustration? What is the nature of this duty? Is it morally defensible to request or, more demandingly, require family members to be present with the unresponsive patient to bear witness to their actual or perceived suffering? If so, on what grounds?

In this paper, we examine several possible reasons that might justify the view that people need to bear witness to the suffering^3^ of a seriously ill close family member: (a) to improve their understanding of the patient's clinical situation, especially if they are also serving in the role of surrogate decision‐maker; (b) to provide persuasive evidence that can lead to surrogates’ making medical decisions that the clinical team believes are best for the patient (or, minimally, no longer harmful to the patient); (c) to facilitate a more general moral duty or moral virtue that people have to their family members by being present in their suffering; and (d) to mitigate or “share” the moral and emotional burdens faced by the clinical team. We argue that the first reason is justified, but with less frequency and more complexity than might be assumed. The second reason could be justified but is more often morally unjustified. The third and fourth reasons, we argue, are generally not morally justified. In the end, despite the intuitive pull of the claim that family members have a moral obligation to be present at the bedside of a barely conscious or unconscious patient whom clinicians believe to be suffering, we are largely skeptical that this is an obligation.

## Understanding through Presence

One reason that clinical teams might want surrogates and other close parties to bear witness to patient suffering is so that they can better understand the clinical situation of the unresponsive patient and make medical decisions that are grounded in what the clinical team views as the patient's actual clinical status. Even if family members are not specifically designated as surrogate decision‐makers, often they have influence over, or are helpful to, the surrogate decision‐makers. While it is true that surrogate decision‐makers and family members can be informed about the clinical facts of the case via a phone call, a video call, or written communication, understanding and appreciating the “whole” of the patient's situation often involves a cognitive and affective component that may involve seeing, breathing, and experiencing it. As some of us have expanded on elsewhere, there is an important type of moral knowledge that comes from experience.^4^ Witnessing a person's suffering is itself an experience that generates beliefs and perspectives (knowledge) that might not be present without that experience. In the book *Moral Knowledge*, the philosopher Sarah McGrath gives several examples of moral knowledge gained from experience, including how Einstein, a pacifist, came to see how war could be just after witnessing the horrors of the Nazi era.^5^ Similarly, in *Epiphanies: An Ethics of Experience*, philosopher Sophie Grace Chappell writes about moral insights or “epiphanies” that come “not by argument or deduction, but by our directly and immediately seeing or otherwise experiencing” them.^6^ Chappell recounts how the philosopher Peter Singer came to see the wrongness of animal suffering inflicted by humans, not through philosophical argument, but through something like an epiphany in the face of witnessing it.^7^

If we follow this line of argumentation and take it to be the case that seeing and experiencing firsthand may enable certain forms of knowing that are not possible only by hearing someone else's report of a situation or experience, then seeing and appreciating the situation of the patient, including the suffering they are undergoing, will invariably generate access to an otherwise‐unattainable type of knowledge for surrogates and other caring parties. This, in turn, puts surrogates in a better position to exercise their moral obligation to make medical decisions that are responsive to the patient's actual (and not merely imagined) clinical status and aligned with the patient's interests and values, as communicated prior to becoming unresponsive. We might call this an “epistemic justification” for wanting family members to bear witness: bearing witness gives them important knowledge to help them enact their duties as surrogate decision‐makers or as decision helpers—knowledge without which they might make decisions that do not reflect what the patient would have wanted for herself.

This justification is, on its face, ethically defensible. There are, however, two complications. The first is that this argument works only in cases where it is necessary for the relevant parties to be at the bedside to “get it”—where this information about the nature and extent of patient suffering relative to benefit could not be conveyed to them and appreciated by them via other means. Determining whether witnessing is necessary in a given case, however, is not an easy task, and it would be presumptuous to require it for a surrogate to discharge their duties. Understanding is a matter of degree, and so the moral importance of witnessing suffering will depend on the extent to which a surrogate understands the suffering of the patient and the whole of their situation independently of observation. In some cases, witnessing may improve a surrogate's understanding and put them in a better position to make decisions that are aligned with the patient's interests and values, but the surrogate may already have a sufficient degree of understanding to responsibly exercise their duties as surrogate decision‐maker. In other cases, seeing may be critical to understanding the clinical picture and the patient's situation, making witnessing closer to a moral obligation requisite for discharging one's responsibility qua surrogate. For example, if a patient has recently suffered an acute, irreversible neurologic injury resulting in a coma and her surrogate decision‐maker has not seen her in this condition, the knowledge gained by seeing her might be more morally important for surrogate decision‐making than if the injury had occurred long ago and the surrogate had known the patient in that state for a prolonged period.

The second issue is that, although there may be epistemic value in seeing suffering firsthand, the experience can also distort a family member's or surrogate's understanding of the clinical situation.^8^ Take, for instance, a patient who is in a state of unresponsive wakefulness syndrome, formerly referred to as a “vegetative state.” In this state, patients may grimace or posture as a reflex rather than as a meaningful response to external stimuli. Family members or surrogates who witness this may attribute a type of conscious, experiential suffering to the patient that is not, in fact, occurring and therefore make decisions based on inaccurate interpretations of the patient's clinical presentation.^9^ Or imagine a nurse asking a family member or surrogate to come to the hospital to see a patient's unstageable bedsore, hoping that the stench and graphic image will give the family member the “real medical picture.” Research in moral psychology has demonstrated that the “disgust factor” can trigger strong emotional reactions and moral judgments.^10^ Concerned parties might be triggered by the disgust factor and overestimate the suffering of the patient. In this latter case, if the family member acquiesces to a transition to comfort care, the reasoning would not be because they have a better understanding of the clinical details, such as the wound's irreversibility or the increased infection risk that accompanies such a sore, but because what they saw disgusted them. Such intentions and actions on the part of the treating team potentially threaten appropriate surrogate decision‐making, rather than improving it. This is not to say that having surrogates witness the suffering of a patient always distorts their judgment, but one can imagine cases where it has precisely this effect. The reality is complex and case specific, and, indeed, witnessing may sometimes both improve understanding of the patient's situation in some ways and distort it in others.

## Getting Surrogates to Make the “Right” Choice

Sometimes, wanting surrogates to see the diminished condition or suffering of a patient is intended not primarily to increase epistemic advantage for patient‐centered decision‐making but, rather, to steer surrogates toward particular choices that the clinical team believes are in the patient's interest (but that the decision‐maker is not making). In other words, clinicians might want surrogate decision‐makers to witness an unresponsive patient's real or perceived suffering to nudge^11^ them—typically toward de‐escalation of particularly invasive or life‐sustaining interventions. Clinicians might want family members to see a resuscitation attempt with its brutality and broken ribs so that they agree to a do‐not‐resuscitate order. Or they might want family members to see bedsores or gangrenous limbs to agree to stop treatment that the clinical team believes is no longer beneficial to the patient.

Unlike the desire to inform and educate family members so that they better understand the situation and are well equipped to engage in the process of good surrogate decision‐making, the operative desire here is to influence toward a particular decision. This justification for having family members witness patient suffering is more ethically problematic. As Robert Veatch argues, expertise in matters of science and medicine do not generalize to expertise in value judgments.^12^ Clinicians have expertise about the medical facts of the patient's situation, but they are not authorities on what would be best for a given patient, because such a determination fundamentally involves judgments of what is valuable for or to the patient, and these values may well be unpredictable or idiosyncratic. Indeed, patients are permitted to make “bad” choices—such as decisions that are contrary to medical advice or unduly risky—so long as they fully understand, appreciate, and can offer reasons as to why they are selecting them. That being said, clinicians’ perspectives on suffering should not be outright dismissed. Clinicians’ repeated exposure to patients who are seriously ill and dying can provide its own kind of experiential moral insight that can be valuable, even if it is not definitive. While clinicians are not experts in a given patient's values and priorities, they are often acutely aware of the burdens imposed by medical interventions and the limits of what medicine can meaningfully offer. These views should be held with humility and offered as a part of a collaborative process of decision‐making rather than as a substitute for the surrogate's role of interpreting the patient's values and wishes.

However, there may be some cases in which the desire to have family witness the suffering of a patient to influence their decisions in a particular direction *could be* more clearly ethically defensible. Suppose that the clinical team has good information about what the patient's values and wishes were before she became incapacitated and that the medical decisions that the surrogate decision‐maker is making are clearly not in line with those, even though the clinical team has attempted to discuss and communicate this to the family members involved with decision‐making. In this type of case, where there is a clear choice for the patient that is most consistent with the patient's previously expressed capable wishes, surrogate decision‐makers are not behaving consistently with their obligation to exercise substituted judgment (to make decisions that the patient would have made for herself, were she able to do so). In such a case, clinicians may justifiably ask family members to bear witness to the patient's suffering in an attempt to influence choice in a particular direction (in line with the patient's expressed wishes). As a concrete illustration, imagine that a patient has an advance directive declining prolonged intubation but a surrogate decision‐maker is resisting terminal extubation. After witnessing the patient's visible discomfort despite sedation, the surrogate may become more open to extubation, thus respecting the patient's advance directive.

The situation becomes more complicated, however, when the surrogate decision‐makers are making choices that the clinical team believes go against the patient's interests (rather than the patient's expressed wishes). Here, more subjectivity enters the picture—who gets to determine what is in the patient's interests and on what grounds it is? The less clear it is what is in a patient's interests, the less ethically acceptable it is for clinicians to attempt to have decision‐makers witness the patient's condition to influence their decisions in a particular direction. Under such uncertainty, even identifying that condition with “suffering” raises the questions of what the patient's interests are and what the nature of suffering is. One clear exception here, however, is the phenomenon in which a surrogate attempts to decline appropriate palliation on the patient's behalf. It is an unambiguous right of all patients to have their pain appropriately managed, and clinicians must disregard surrogates’ requests that appropriate pain medication be withheld. Consent is not required to administer appropriate palliation in the face of surrogate refusal if a patient communicates pain or visibly experiences it. As a result, the suggestion that witnessing suffering is required to generate the appropriate clinical outcome—namely, pain management—is incorrect. Pain ought always to be managed, irrespective of what the surrogate sees or decides.

## Sharing and Offloading the Moral Burden

Clinicians must frequently bear witness to the actual or perceived suffering of unresponsive patients in ways that are emotionally and morally taxing and may even create moral distress. In such cases, caregivers’ absences at the bedside may amplify this burden, as the responsibility of witnessing suffering falls solely on the clinical team. This dynamic can give rise to a subtle expectation that others outside of the treating team should be present to “share” the burden of witnessing, as if their presence validates or helps offset the clinicians’ moral distress. The act of witnessing might be framed as a form of moral engagement that distributes the emotional and ethical weight between the patient's family members and the clinical team. This could be thought of as a form of communal moral burden, through which the moral burdens are mitigated, leveled out, or shared. In these cases, clinicians’ desire for a redistribution of this moral burden may also reflect a deeper distress rooted in a sense of limited agency in clinical decision‐making and an inherent tension between clinical care and the foundational obligations of the medical profession, namely, to provide beneficial treatment and avoid harmful treatment. In those moments, the clinician's role may feel misaligned with values central to medical ethics, which may intensify the urge to seek out shared moral responsibility or accountability from the patient's family members.

The impulse to offload some of the moral burden and shared accountability onto a patient's absent family members is likely multifactorial, complex, and may sometimes stem from significant and prima facie understandable internal conflicts about professional obligations. This very human impulse raises ethical concerns. The presence of others at the bedside should be grounded in the patient's interests and the surrogate's obligations, not in the needs of clinicians to alleviate their own emotional or professional burdens. In fact, pressuring family members to be present risks instrumentalizing family members and hoisting additional moral and emotional burdens on them. There may be good reasons to work to alleviate the moral and emotional stress and burden experienced by the clinical team. Alternative mechanisms to address this distress, including institutional ethics support, peer discussions, or professional counseling should be employed.

One final point bears mentioning, though. Sometimes the clinical team might want these individuals to be present to confirm or contextualize the interventions they are performing as in line with the patient's wishes and values. This may appear as a desire to share or offload moral burden (and may indeed have the secondary effect of such emotional offloading) but is really a legitimate desire for shared decision‐making with the surrogate and anyone else who may be supporting her.

## Witnessing Suffering as a Virtue or Duty

Some clinicians (or others) may hold that, in certain cases, it is not enough for a surrogate decision‐maker to fulfill the duties of that role. They may believe that, when a surrogate is the patient's family member, they should bear witness to the patient's suffering. This view often appeals to broader notions of moral virtue or obligation. On this view, witnessing is seen as an expression of care, love, solidarity, and moral fidelity to the patient. It reflects the belief that presence at a time of suffering is not just helpful but inherently “the right thing to do.”

Yet this argument invites scrutiny for several reasons. First, if there were such a duty or good (virtue), it is not clear that it would persist when the patient is unconscious or unable to perceive the presence of others. Perhaps there is a theory of virtue capable of explaining the positivity of behaviors in terms of their excellence within a framework of relationships that reflects the nature of our dependency and vulnerability as human beings.^13^ Something like this was perceived by Harriet McBryde Johnson in her justly famous debate with Peter Singer, who had argued to her that taking care of a permanently unconscious patient was “a bit—weird.” “No,” she replied. “Done right, it could be profoundly beautiful,” because it can reflect an activity of “unconditional commitment.”^14^ Like all virtue theories, this view focuses primarily on the agency of the actors and not on the recipients of the actions. Those who interpret the principle of beneficence as requiring a material benefit to accrue to the recipient of the action for the action to have moral value could argue that such a virtue might not apply to cases in which patients are unresponsive and wholly or largely unaware (the focus of this paper). Second, it is not clear that it would persist when the act of witnessing risks significant emotional harm to those tasked with bearing witness. In short, even if it were the case that intimates have a moral obligation to provide presence and attention, such duties are not absolute and indeed are defeasible. They must be balanced against these individuals’ capacities, their vulnerabilities, and the patient's own values and preferences.

Framing witnessing as a moral duty risks overextending the ethical expectations placed on family members. Moreover, there is a danger of moralizing absence in ways that fail to account for the complex realities that individuals face, such as emotional exhaustion, unresolved interpersonal conflict, or logistical barriers. The desire for bedside presence should focus on the goods of meaningful engagement (with the patient, the whole existential situation, or the clinical team) rather than become a burdensome expectation. Perhaps shifting expectations of the surrogate would benefit the team and offer a more justifiable scheme.

## Moral Reckoning

A more ethically fraught motivation for requiring supportive individuals to witness suffering can arise from a desire for moral leveling or moral reckoning. Moral reckoning can involve coming to terms with one's own moral accountability. When clinicians are compelled to provide treatments that they perceive as prolonging suffering or lacking benefit, they may feel that those requesting it should confront the consequences of these decisions. Statements along the lines of “If I have to see this every day, so should they” can reflect a frustration that borders on a desire for the family to experience a moral reckoning or even retribution. Clinicians’ distress can be further amplified by the reality of functioning in an environment that requires careful allocation of their time, attention, and emotional energy. Spending prolonged periods at the bedside of unresponsive patients can feel burdensome, particularly in the absence of family. Thus, the desire for a patient's family members to witness the patient's condition may reflect the clinician's need to justify a continued investment of their time and emotional effort.

The desire for others to undergo moral reckoning for their perceived wrongs has been well‐documented in the psychological literature. For example, Jessica Salerno and Michael Slepian explore how individuals use punitive actions, such as exposing others’ moral failings, to compel them toward moral accountability.^15^ Similarly, research by Brock Bastian and colleagues highlights how moral outrage can motivate individuals to demand retributive justice, especially when the outrage is combined with feelings of dehumanization and disgust about the perceived offenders.^16^ Within clinical settings, moral outrage may manifest as a desire to force surrogate decision‐makers to confront the moral weight of their decisions by witnessing the suffering that their choices prolong.

While the instinct to seek moral reckoning from a patient's family members may be rooted in professional and logistical causes of distress, requesting that a surrogate witness suffering for this purpose is ethically indefensible. The demand for carers at the bedside must not be construed as a punitive measure or a means to assign blame. Requiring such individuals to endure the distress of witnessing suffering as a form of payback or moral accountability distorts the nature of the clinician‐surrogate relationship and can undermine trust not only within this dyad but also in the health care system more broadly.

Given the clinical, moral, and emotional complexity involved in caring for unresponsive patients, it is understandable that clinicians may feel an instinctive desire for a patient's family members to be present at the bedside. This impulse may arise from a need to share the burden of witnessing the patient's perceived or actual suffering, to validate the time‐intensive clinical and emotional care the clinician is providing, to reconcile a conflict about professional duties, or to restore a sense of moral and clinical agency in the context of limited clinical options. But clinicians must approach surrogate absence with a spirit of compassion and curiosity rather than judgment. While it is appropriate to invite surrogates to witness and engage in the patient's care and it may (depending on the motivation behind it) even be a defensible extension of how to resolve intractable dilemmas in medical decision‐making, this invitation should be framed as an opportunity for understanding, not a demanding imposition or guilt‐laden obligation.

For clinicians who feel a strong urge to implore absent family members of unconscious patients to be present at the bedside, it is essential to reflect on the underlying motivations. This reflection can be initiated by the clinical team, or it can be facilitated by an ethicist. Is this desire rooted in the patient's interests? Is it based on a concern about appropriate surrogate decision‐making rooted in insufficient understanding of the patient's clinical status? Is it an attempt to share or offload a moral burden? Is it a call for validation of the difficult clinical and emotional work involved in caring for a minimally conscious patient? Or is it an attempt at moral leveling? Clinicians should be encouraged to ask themselves whether the presence of a family member would serve a therapeutic, communicative, or otherwise ethically defensible purpose, or whether it risks exploiting the family member's presence for other, less ethically defensible (or even overtly indefensible) reasons.

In the end, we remain largely skeptical of the view that family members have an uncomplicated moral obligation to be present at the bedside of a barely conscious or unconscious patient who clinicians believe to be suffering. At the very least, scrutiny of the instinct to blame those who are not present is needed.
